# TCAD simulation study of heavy ion radiation effects on hetero junctionless tunnel field effect transistor

**DOI:** 10.1038/s41598-024-58371-6

**Published:** 2024-04-01

**Authors:** K. Aishwarya, B. Lakshmi

**Affiliations:** 1 School of Electronics Engineering, Vellore Institute of Technology, Chennai, India; 2 Centre for Nano-Electronics and VLSI Design and School of Electronics Engineering, Vellore Institute of Technology, Chennai, India

**Keywords:** HJLTFET, Heavy ion radiation, SiGe, InAs, GaN, GaAs, Electrical and electronic engineering, Materials for devices

## Abstract

Semiconductor devices used in radiation environment are more prone to degradation in device performance. Junctionless Tunnel Field Effect Transistor (JLTFET) is one of the most potential candidates which overcomes the short channel effects and fabrication difficulties. In this work, 20 nm JLTFET is proposed with Silicon in the drain/channel region whereas source uses different materials, Silicon Germanium (SiGe), Gallium Nitride (GaN), Gallium Arsenide (GaAs), Indium Arsenide (InAs). The device performance is examined by subjecting it to heavy ion radiation at a lower and higher dose of linear energy transfer (LET) values. It can be seen that the most sensitive location is the source/channel (S/C) interface for SiGe, GaN and GaAs whereas the drain/channel (D/C) interface for InAs. Further analysis is carried out at these vulnerable regions by matching I_ON_ of all materials. The parameters, transient peak current (I_peak_), collected charge (Q_C_), threshold voltage shift (ΔV_th_) and bipolar gain (β) are extracted using transient simulations. It is observed that for a lower dose of LET, I_peak_ of SiGe is 27% lesser than InAs and for higher dose of LET, SiGe shows 56% lesser I_peak_ than InAs. SiGe is less sensitive at lower and higher dose of LET due to reduced ΔV_th_, tunneling and electron density.

## Introduction

Electronic devices when used in the nuclear power industries, space systems and national security systems are mostly vulnerable to radiation environments. Under a radiation environment, the devices change their electrical parameters causing device failure^1^. The radiation effects on these semiconductor devices pose a serious threat to electronic industries. Many devices are being explored to mitigate the effects of radiation in semiconductor environments. Under radiation exposure, MOSFET causes radiation induced damage to the device due to the trapped charges on the dielectrics causing variations in the device characteristics^2^. Pejovic et al. studied that for a p-channel MOSFET, increased radiation dose causes threshold voltage shift changing the sensitivity of the device^3^. The radiation analysis based on Silicon carbide (SiC) MOSFET revealed that the radiation induced trapped charges are lesser than Silicon based MOSFET making SiC MOSFET resistant to harsh radiation of more than 100 K rad^4,5^. Kumar et al. concluded that n-channel Transparent Gate Recessed Channel (TGRC) MOSFET shows more sensitivity toward radiation than conventional MOSFET^6^. The vertical double-diffused MOSFET under heavy ion strike produced more interface trapped charges causing degradation to device performance^7^.

In a radiation study by Hubert et al., FinFET based devices show more resistance towards ionizing radiation due to the lesser sensitivity volume^8^. The dose radiation effects on FinFET explored threshold voltage shift for the value of dose higher than 300 K rad (SiO_2_)^9,10^. The total ionizing dose (TID) effect on Si based FinFET and SiGe based FinFET considering the fin width, bias and orientation was discussed^11,12^. Tunnel FET (TFET) was developed as an alternative to MOSFET to be used in ultra-low power applications^13–15^. The radiation study on Silicon based TFET device shows a little degradation towards radiation and is found suitable for space applications^16^. Dubey et al. studied the gamma radiation effect on Silicon on Insulator (SOI) TFET claiming that the device shows excellent resistance in ON state^17^. Yan et al. explored SET and TID effects on Ferroelectric TFET (FeTFET) providing good radiation resistance^18^. The heavy ion radiation study on L-shaped TFET (LTFET) by considering the effects of LET and voltage bias shows the device is sensitive to radiation^19^.

It is further noted that Junctionless FET (JLFET) based devices like Junctionless Double Gate Radiation Sensitive FET(JLDGRADFET) have more sensitivity when the threshold voltage of the device increases^20^. It is further noted in the radiation study by Wang et al. on Junctionless Dual Material Double Gate MOSFET (JLDM-DGFET) to have excellent radiation hardness by exploring bipolar gain and collected charge^21^. A study on Ge-Junctionless CMOSFET under X-ray radiation attack shows a larger shift in threshold voltage^22^. The heavy ion radiation study on Graded Channel Junctionless Double Gate FET (GC-JLDGFET) shows reduced collected charge after an ion strike with reduced I_peak_^23^. The heavy ion radiation effect on silicon based Junctionless Accumulation Mode Double Gate Transistor (JAM MOSFET) demonstrates better radiation hardness at lower dose of LET values^24^.

Various literature extensively studied the behaviour of hetero structured devices in a radiation environment. Weatherford et al. presented an exhaustive survey on the historical perspective on radiation effects on III–V devices^25^. McMorrow et al. analysed the transient response of the III–V field effect transistor to heavy ion radiation^26^. The single event effect (SEE) in III–V circuits and methods to mitigate their impact is explored^27^. It is found that SiGe Heterojunction Bipolar Transistors (HBT) have a lot of potential for functioning in a range of harsh radiation environments^28^. The simulation model of SiGe HBT was studied providing a theoretical framework for additional radiation hardening^29^. The proton irradiation effects on AlGaN/GaN High Electron Mobility Transistors (HEMT) are studied in the literature^30^. The gamma irradiation impact for determining the device performance and reliability on electronic carrier transport in AlGaN/GaN HEMT is analysed^31^. The radiation resistance of AlGaN/GaN and InAlN/GaN HEMTs and GaN–based LEDs to different types of ionizing radiation is reviewed^32^. The Total Ionizing Dose (TID) Effects in SiGe MOS FinFETs are investigated under different device bias conditions^11^. The gate bias and length dependences of TID Effects in InGaAs FinFETs on Bulk Si are extensively evaluated^10^. The radiation-induced soft errors have been assessed for Si FinFET, III–V (InAs) FinFET, and III–V (GaSb Source/InAs Channel-Drain) HTFET^33^. An excellent anti-radiation performance has been achieved using an N-type TFET with a Si_1−x_Ge_x_/Si hetero-junction in the ultra-shallow N+ pocket region^34^. The Vertical JLFET with the Ge source region obtained an improved radiation hardness^35^.

One of the promising devices that could be used for radiation mitigation is Junctionless Tunnel FET (JLTFET) as it exploits the benefits of TFET and JLFET. TFET acts as a gated PIN diode which works on the principle of band to band tunneling. The device shows excellent characteristics in terms of superior OFF state current, lower subthreshold slope (SS) and higher switching ratio (I_ON_/I_OFF_) making it suitable for low power electronics. The device has excellent gate control in the channel and does not have bipolar transistor avoiding the deposited charge amplification making it suitable for radiation prone environment^36,37^. For a smaller channel device, the presence of ultra sharp junctions causes variation in doping concentration giving rise to thermal budget. The junctionless transistor is normally an accumulation mode device with the doping concentration of channel being the same as that of source and drain. The accumulation mode device normally shows better short channel characteristics than conventional inversion mode devices. The usage of same doping concentration throughout the device eradicates the concentration gradient thereby relaxing thermal budget to a greater extend. The fabrication is also made simpler as there are no sharp junctions^38^. JLTFET works by inheriting the advantages of both TFET (steeper SS) and JLFET (increased drive current). It avoids the physical doping of the source, drain and channel region and is free from random dopant fluctuation boosting the immunity towards short channel effects (SCE). The fabrication of JLTFET is simple as there is no metallurgical junction^39,40^. In our previous work, homojunction based JLTFET is investigated under heavy ion radiation^41^. The DC and analog characteristics of JLTFET are improved by using III–V materials which could be used as hetero JLTFET (HJLTFET)^42–44^. Since the study of heavy ion radiation on HJLTFET was not discussed earlier in the literature, our work throws light on the impact of radiation sensitivity for HJLTFET.

In this work, the heavy ion radiation study is carried out on III-V HJLTFET by interfacing III-V with group IV semiconductors. The drain and channel regions are fixed as silicon and various source side materials like Silicon Germanium (SiGe), Gallium Nitride (GaN), Gallium Arsenide (GaAs), Indium Arsenide (InAs) are chosen to form HJLTFET. The radiation-sensitive metrics, collected charge (Q_C_), transient peak current (I_peak_), threshold voltage shift (V_th_) and bipolar gain (β) are extracted for different linear energy transfer (LET) values. The paper is organized as follows: section “Device description and simulation methodology” discusses HJLTFET device structure and simulation methodology. The next section covers the results and discussion. Last section presents the conclusion.

## Device description and simulation methodology

### Device structure

**Figure 1:**
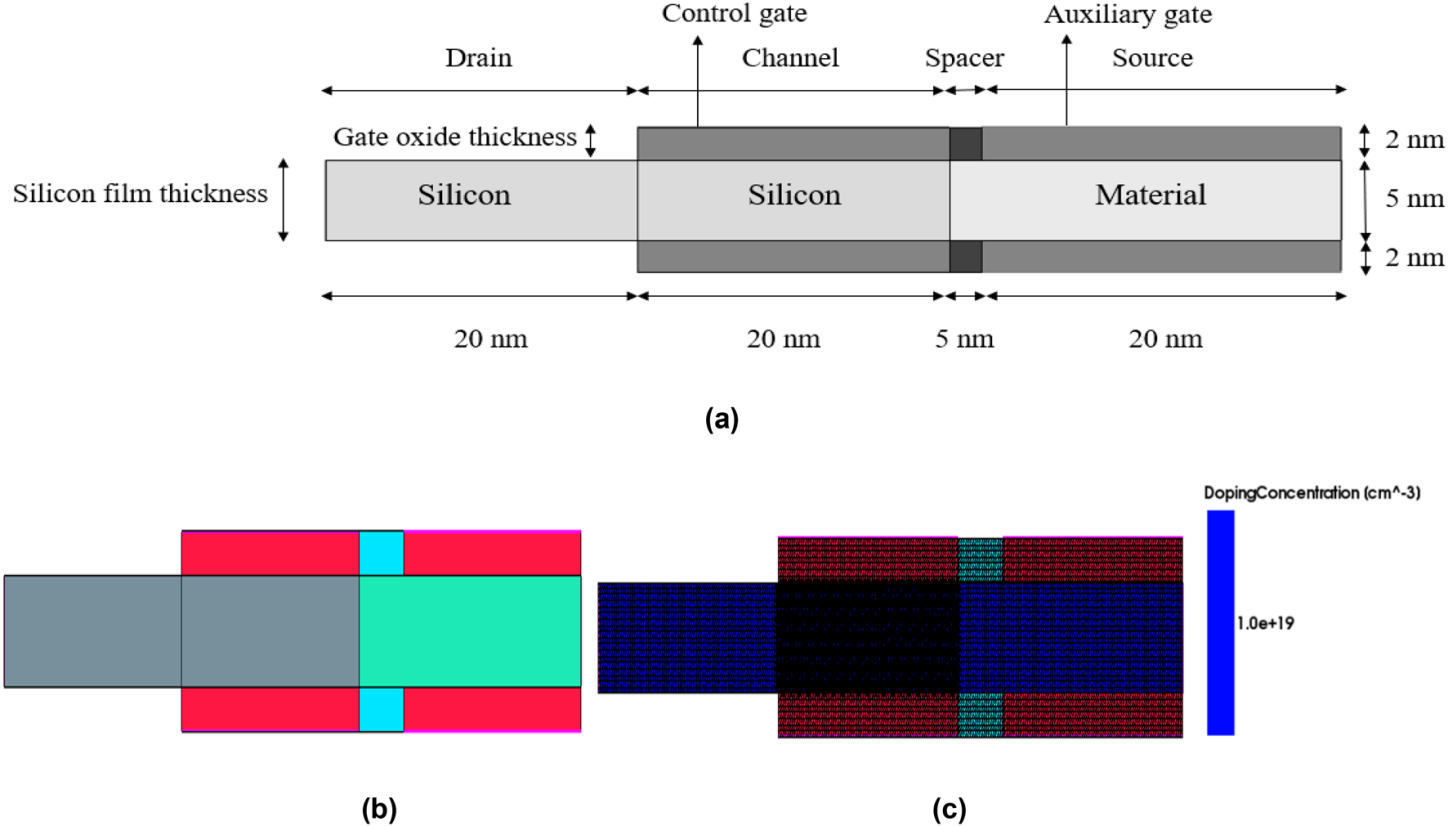
(**a**) Schematic structure of HJLTFET. (**b**) Simulated structure of HJLTFET (without doping). (**c**) Meshed structure of doped HJLTFET.

Sentaurus TCAD simulator is used for this study^45^. The schematic, simulated and meshed structure of HJLTFET is shown in Fig. 1a–c. It has two gates namely the control gate (CG) and auxiliary gate (AG) with Silicon in the drain and channel region and different materials are taken for the source region. The source side material determines the maximum value of I_ON_ since it affects ON-state tunnelling^42^. The materials chosen for the source are a mixture of narrow and wide energy band gaps such as SiGe, GaN, GaAs and InAs. These materials are chosen since wide energy bandgap devices help to reduce the ambipolar current whereas narrow bandgap devices exhibit higher I_ON_^46,47^. The simulation parameters are displayed in Fig. 1a. The simulated device is comprised of a silicon channel and drain with a high doping concentration of 1 × 10^19^ cm^−3^, a channel length of 20 nm, a silicon film thickness of 5 nm, source/drain extensions of 20 nm, isolation between CG and AG of 5 nm, and a gate oxide thickness of 2 nm. The insulator used here are Hafnium Oxide (HfO_2_) and Silicon Dioxide (SiO_2_). HfO_2_ is a high ‘k’ dielectric which is used as gate oxide and SiO_2_ with low-k is used as spacer oxide. As it could be noted from the literature, a combination of low and high ‘k’ dielectric material above the substrate helps in improving DC characteristics like higher ON current (I_ON_) and a lower leakage current^48,49^. The devices used in the study are represented as Si/Material which means Silicon is used in the drain and channel region whereas hetero materials are used in the source region. The notation for the devices used in this study is Si/SiGe, Si/GaN, Si/GaAs and Si/InAs. The structure is calibrated with the work function (WF) of CG and AG fixed to 4.3 eV and 5.93 eV respectively^39^.

**Figure 2:**
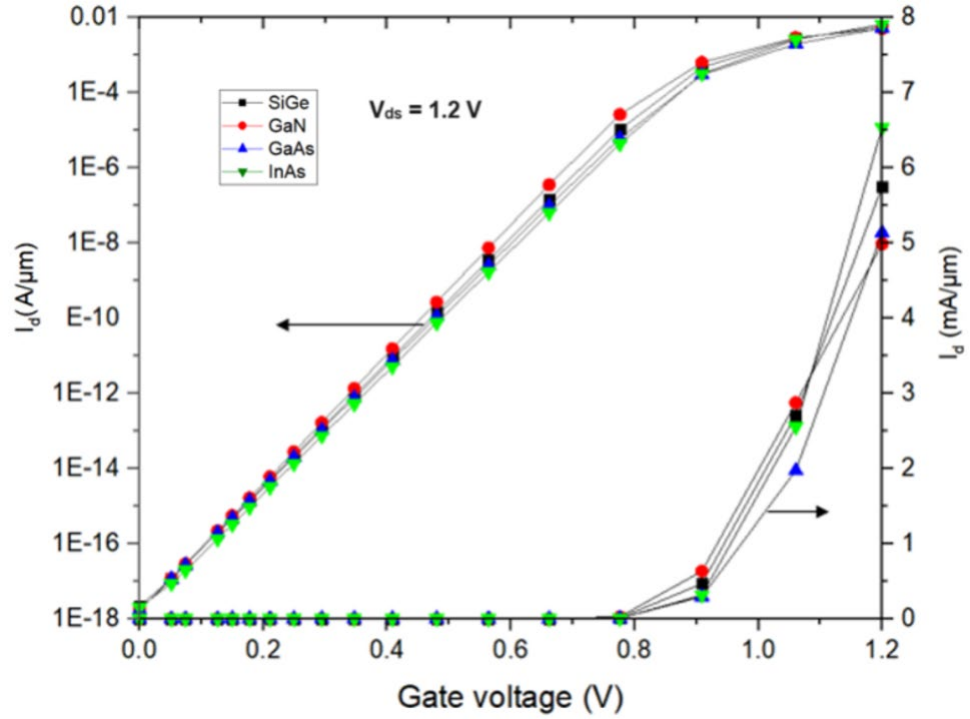
I_d_–V_g_ characteristics of HJLTFET.

By taking the effects of the electric field on mobility and velocity saturation into account, the device simulator incorporates the required models for device simulation in the physics section. The Shockley–Read–Hall (SRH) recombination model and the Hurkx model with Fermi statics for the band-to-band tunnelling (BTBT) model are all employed. Due to the high doping concentration, band gap narrowing is also included. The quantization effects are considered using density gradient model. The heavy ion model is employed to simulate the heavy ion strike. The I_d_–V_g_ characteristic of HJLTFET with various source materials is plotted by matching the I_OFF_ as shown in Fig. 2. The linear and log scale of the drain current is shown in left and right axis respectively. In this study, a supply voltage of 1.2 V is used. The threshold voltage (V_T_) and SS for HJLTFET are shown in Table 1.

**Table 1:** V_T_ and SS comparison of HJLTFET with various source materials.

Source materials	${\varvec{V}}_{{\varvec{T}}}$(V)	SS (mV/dec)
SiGe	0.361	60.7
GaAs	0.519	61.1
GaN	0.607	60.8
InAs	0.635	61

**Figure 3:**
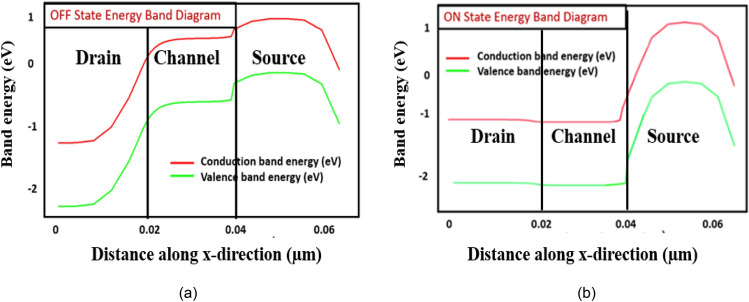
Energy band diagram of HJLTFET in the (**a**) OFF state (V_ds_ = 1.2 V, V_gs_ = 0 V), (**b**) ON state (V_ds_ = 1.2 V, V_gs_ = 1.2 V).

The energy band diagram of HJLTFET in ON and OFF state is shown in Fig. 3a,b. It is found from Fig. 3a that the tunneling barrier between the source and channel is very large giving rise to the negligible electron tunneling in OFF state. The device is then turned on by applying a gate voltage narrowing the barrier between the source and channel of the device as shown in Fig. 3b.

### Simulation methodology

In this study, radiation analysis is carried out after matching I_ON_ of all materials by striking heavy ions at varying LET values where LET stands for energy lost per unit length and is given^50^ in MeV/mg/cm^2^. The parameters, I_peak_, Q_C,_ ΔV_th_ and β are extracted by transient simulations (time period from 0.1 femto to nano seconds). A radiation model with a track length of 1.8 nm and a typical radius of 10 nm at a time of 1 fs is used to simulate the heavy ion strike^45^. The heavy ion strike is made at different locations of the device to find the most vulnerable region. The first step in the simulation procedure is to perform a transient simulation in order to determine the generation rate of electron–hole (EHP) pairs. Based on the total number of additional electrons/holes generated, the carrier continuity and Poisson equations are solved, and transient current and collected charge are ultimately determined^51^. In order to account for the photonic emission, the effect of photons can be modelled using stimulated recombination rate using the Eq. (1).

The stimulated recombination rate is given by,

1 $$ R^{st} (x,\;y) = \mathop \sum \limits_{i} r^{st} \left( {\hbar \omega_{i} } \right)S_{i} \left| {\Psi_{i} (x,\;y)} \right|^{2} $$

where, $\hbar \omega_{i}$ is the stimulated emission coefficient, $S_{i}$ is the photon rate and $\left| {\Psi_{i} (x,\;y)} \right|^{2}$ is the local field intensity.

Equation (2) could be employed to represent the carrier generation rate induced by heavy ions^45^.

2 $$ G \, (l,\;w,\;t) \, = \, G_{LET} (l). \, R \, (w,l). \, T(t) $$

where the functions characterising the temporal and spatial fluctuations of the generation rate are denoted by *T*(t) and *R* (*w*, *l*), respectively.

Equation (3) is used to find the LET generated density, or *G*_*LET*_ (*l*), which has pairs/cm^3^ as its unit.

3 $$ G_{LET} \;(l) = a_{1} + a_{2} + a_{3} e^{{a_{4} l}} + k^{\prime}\left[ {c_{1} \;(c_{2} + c_{3} l)^{{c_{4} }} + LET\_f\;(l)} \right] $$

An exponential function or a Gaussian function can be used to describe the spatial distribution, *R* (*w*, *l*). For this investigation, the Gaussian distribution, described by Eq. (4), is used.

4 $$ R(w,\;l) = \exp \left( { - \left( {\frac{w}{{w_{t} (t)}}} \right)^{2} } \right) $$

where the perpendicular distance from the path is expressed by the radius, w.

*T*(*t*) is again described as a Gaussian distribution as shown in Eq. (5)

5 $$ T(t) = \frac{{2.\exp \left( { - \left( {\frac{{t - t_{o} }}{{\sqrt 2 .S_{hi} }}} \right)^{2} } \right)}}{{\sqrt 2 .S_{hi} \sqrt \pi \left( {1 + erf\left( {\frac{{t_{o} }}{{\sqrt 2 .S_{hi} }}} \right)} \right)}} $$

where t_o_ is the heavy ion penetration time and S_hi_ is the Gaussian characteristic value.

The important metrics, collected charge, deposited charge and bipolar gain are studied for radiation analysis which is similar to our previous work^41^. The drain current is integrated over time to produce Q_C_, and this may be done by using the following Eq. (6)

6 $$ Q_{C} = \mathop \smallint \limits_{0}^{t} I_{d} .dt $$

Equation (7) could be used to determine the deposited charge (Q_dep_).

7 $$ Q_{dep} = LET*t_{Si} $$

where t_Si_ is the silicon film thickness.

β is defined as the amplification of Q_dep_ caused by heavy ion radiation on the device sensitive location which can be found in Eq. (8)

8 $$ \beta = \frac{{Q_{C} }}{{Q_{dep} }} $$

As stated in our previous work^41^, the device will be radiation insensitive if I_peak_ is lesser than I_ON_ which can be found in Eq. (9).

9 $$ I_{ON } \ge I_{peak} $$

## Results and discussion

In this section, the device sensitive location is found for the mentioned hetero materials which are used only at the source side. This is performed by finding the two metrics, I_peak_ and Q_C_. Based on the values of these two parameters, a sensitive location for that device is found. This is repeated for all devices and to have a fair comparison, I_ON_ of all devices is matched by properly tuning the WF of both CG and AG. Then the device is exposed to heavy ion radiation and the device sensitivity is studied for varying values of LET.

### Finding sensitive location on the device

**Figure 4:**
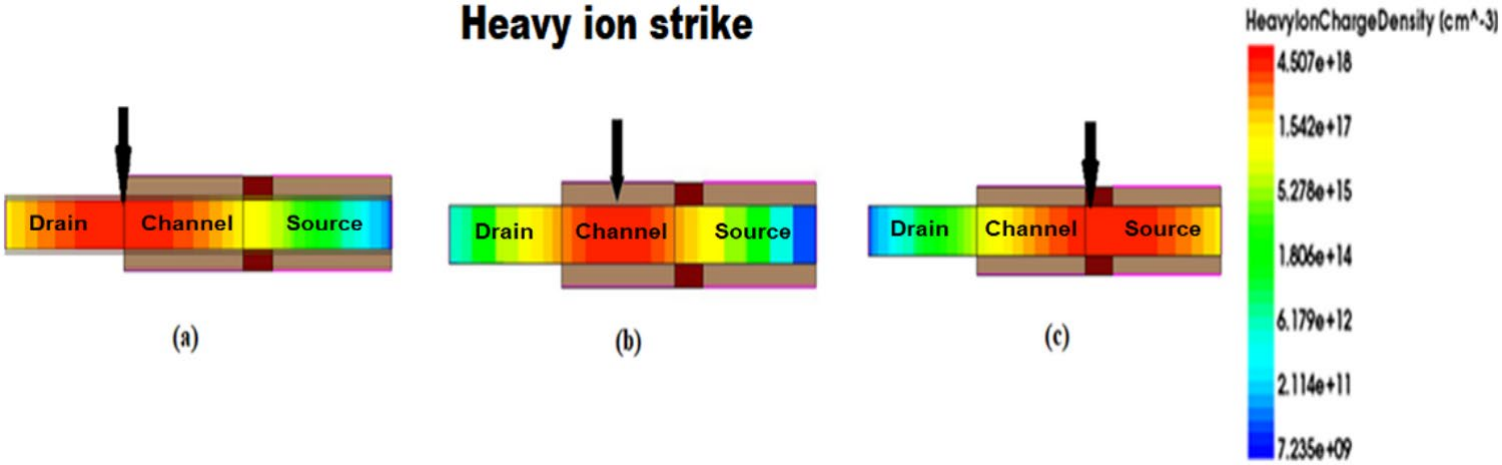
Simulated structure of JLTFET with heavy ions striking at (**a**) drain/channel interface, (**b**) middle of the channel and (**c**) source/channel interface.

To know the effects of heavy ion radiation, HJLTFET is subjected to heavy ion strike and its performance is studied. The heavy ion is made to strike at three different locations namely source to channel interface (S/C), middle of the channel and drain to channel interface (D/C) to know the device’s most sensitive location as shown in Fig. 4.

**Figure 5:**
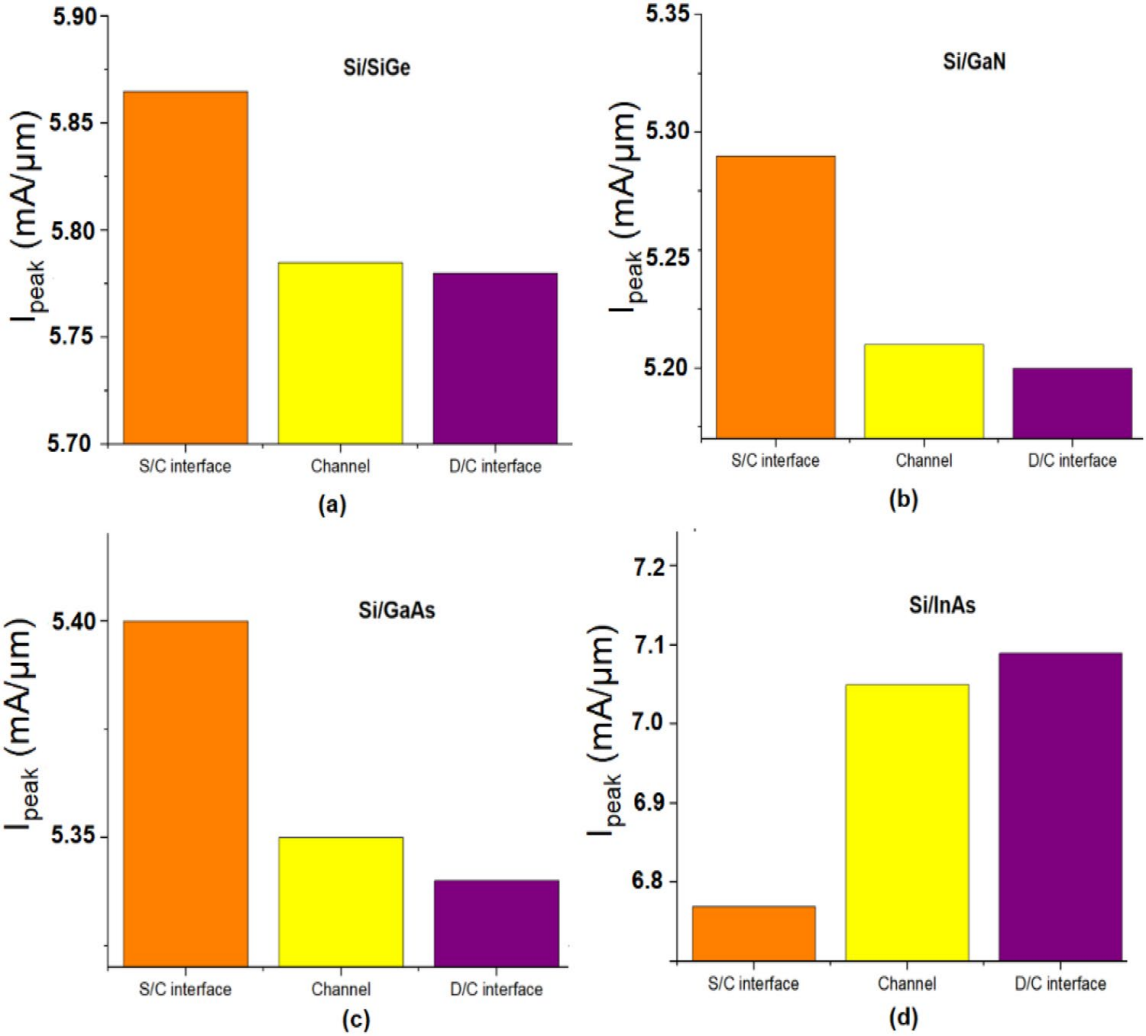
Transient peak current after heavy ion strike at different region of JLTFET (**a**) Si/SiGe, (**b**) Si/GaN, (**c**) Si/GaAs, (**d**) Si/InAs.

**Figure 6:**
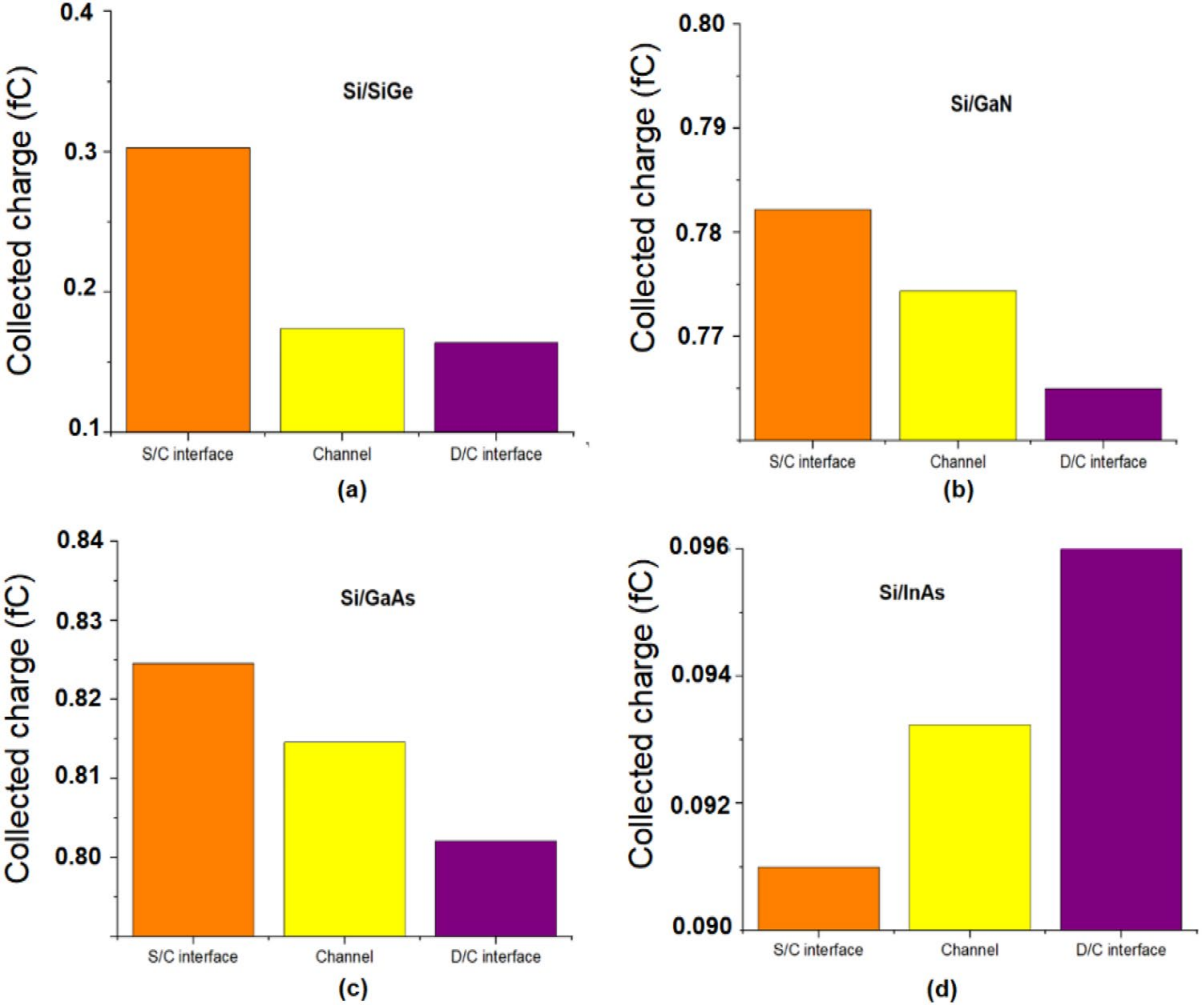
Collected charge after heavy ion strike at different region of JLTFET (**a**) Si/SiGe, (**b**) Si/GaN, (**c**) Si/GaAs, (**d**) Si/InAs.

**Figure 7:**
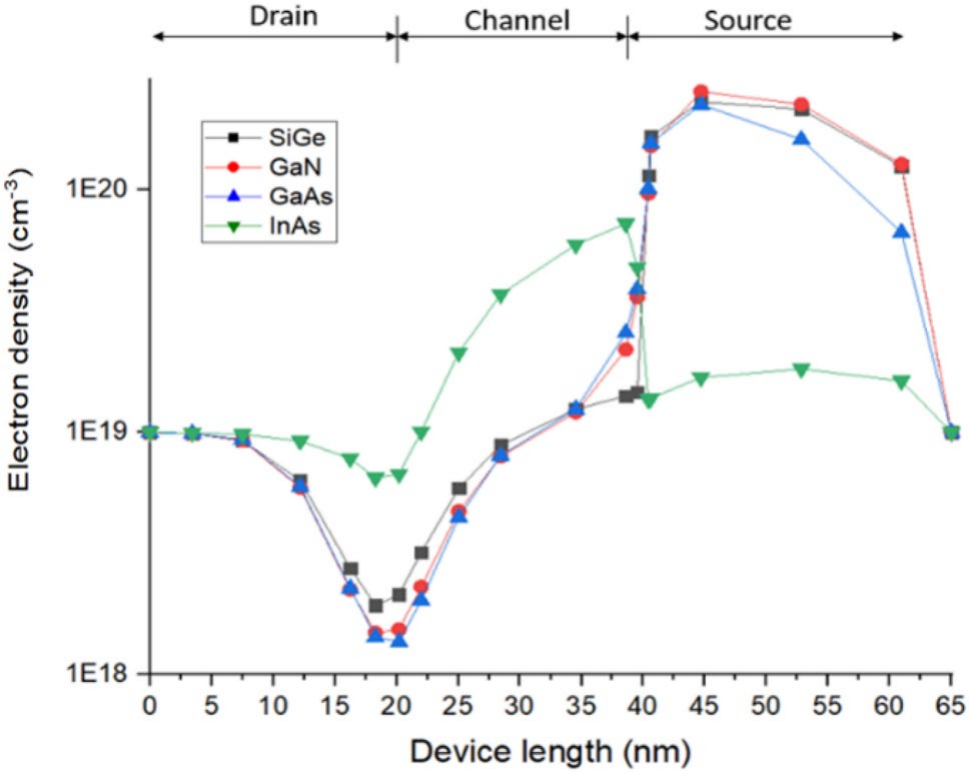
Electron density after heavy ion strike along the device length for different materials.

Using transient simulation, I_peak_ and Q_C_ are extracted for all the different material and is plotted in Figs. 5, 6. From the value of I_peak_ and Q_C_ it can be found that the S/C interface is found to be the most sensitive location as I_peak_ is higher for Si/SiGe, Si/GaN, Si/GaAs based JLTFET and the D/C interface is the most sensitive location for Si/InAs JLTFET. This can be reasoned out with the electron density of the device at different regions which is shown in Fig. 7. From Fig. 7, it can be observed that electron density is higher at the S/C interface for Si/SiGe, Si/GaN, Si/GaAs whereas it is lower for Si/InAs. Hence the radiation sensitive location may be found to be at the S/C interface for Si/SiGe, Si/GaN, Si/GaAs and at D/C for Si/InAs.

### Effect of heavy ion radiation with different materials on HJLTFET

The heavy ion strike is studied for HJLTFET device with different materials, Si/SiGe, Si/GaN, Si/GaAs and Si/InAs. As the S/C interface is found to be the most sensitive location in SiGe, GaN and GaAs, further analysis is carried out at this location whereas for InAs subsequent analysis is carried out only at the D/C interface. For a fair comparison, this study is carried out by matching I_ON_ for all the devices. The LET considered for the study^24,41^ is 1.24 MeV/mg/cm^2^ and 150 MeV/mg/cm^2^.

**Figure 8:**
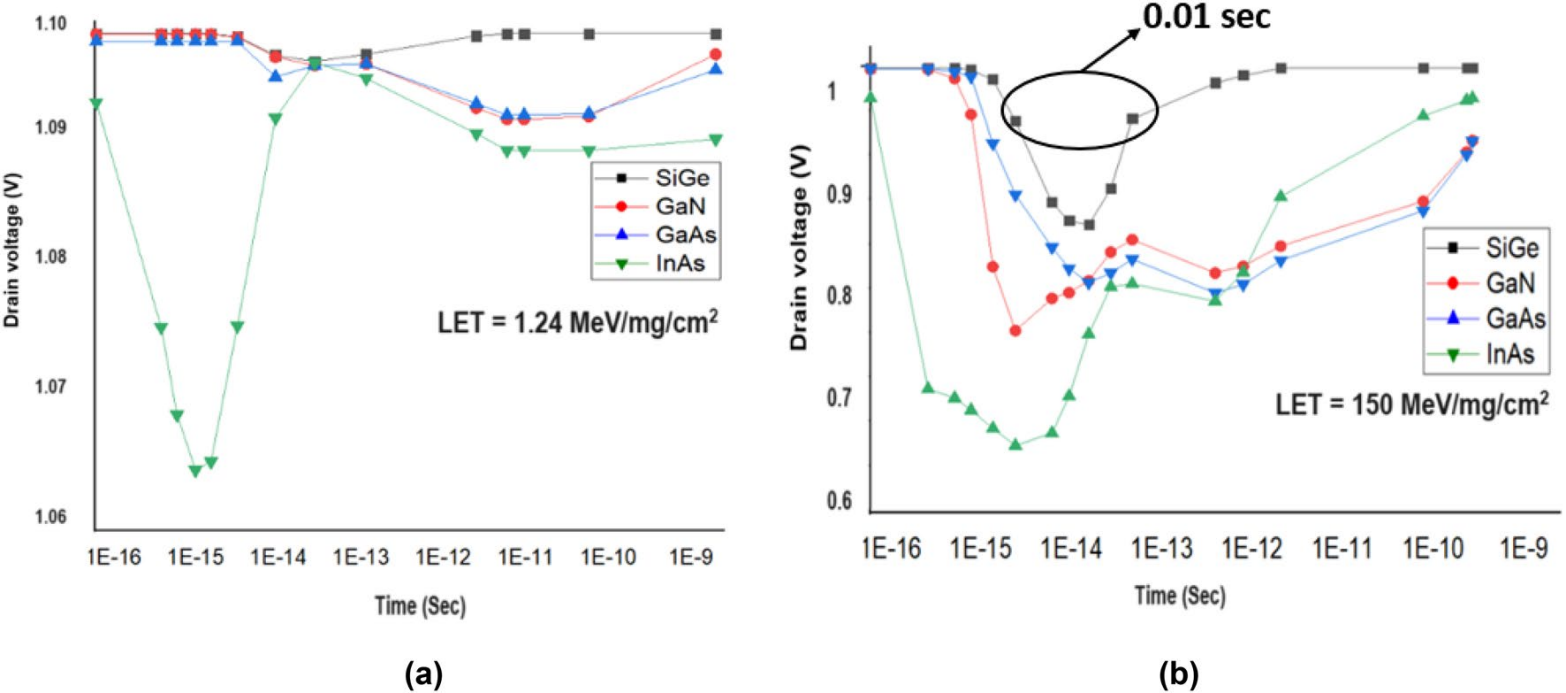
Drain voltage due to heavy ion irradiation for (**a**) lower dose and (**b**) higher dose of LET.

The heavy ions are made to strike at the sensitive location for the mentioned LET values and a change in drain voltage (V_th, SEB_) is observed with respect to different time instants and the change corresponding to a lower and higher dose of LET is plotted in Fig. 8. Figure 8a shows that for a lower dose of LET, SiGe, GaN and GaAs shows higher V_th, SEB_ whereas InAs gives lower V_th, SEB_. V_th, SEB_ can be defined as the minimum drain voltage required to trigger single event burst (SEB) which causes the device failure when heavy ion strikes the device^52,53^. From Fig. 8b it can be observed that for a higher dose of LET, SiGe shows higher V_th, SEB_ than other materials. Since the transient peak time is of very short duration (0.01 s) for SiGe, its sensitivity is less than other materials^21^.

The sensitivity (S) of the device towards radiation is directly proportional to the threshold voltage shift (ΔV_th_) as given by Eq. (10)^17^

10 $$ {\text{S}} = \frac{{\Delta V_{th} }}{D} $$

where D represents Dose.

${\Delta }V_{th}$ can be defined as the absolute difference between the threshold voltage without SEB (V_th, No SEB_) and with SEB (V_th, SEB_) and is given by Eq. (10)

10 $$ {\Delta }V_{th} = \left| {{\text{V}}_{{{\text{th}},\;{\text{No}}\;{\text{SEB}}}} {-}{\text{ V}}_{{{\text{th}},\;{\text{SEB}}}} } \right| $$

**Figure 9:**
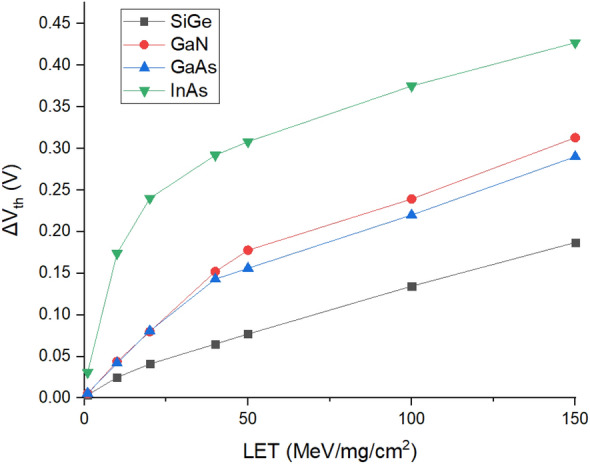
Threshold voltage shift (${\Delta }$V_th_) for various values of LET.

The variation of ${\Delta V}_{{{\text{th}}}}$ with respect to LET values for the different materials is plotted in Fig. 9.

It can be seen from Fig. 9 that ${\Delta V}_{{{\text{th}}}}$ is lesser for SiGe followed by GaAs, GaN and InAs. Thus, the sensitivity of SiGe based HJLTFET towards heavy ion radiation is less followed by GaAs, GaN and InAs which obeys Eq. (9).

**Figure 10:**
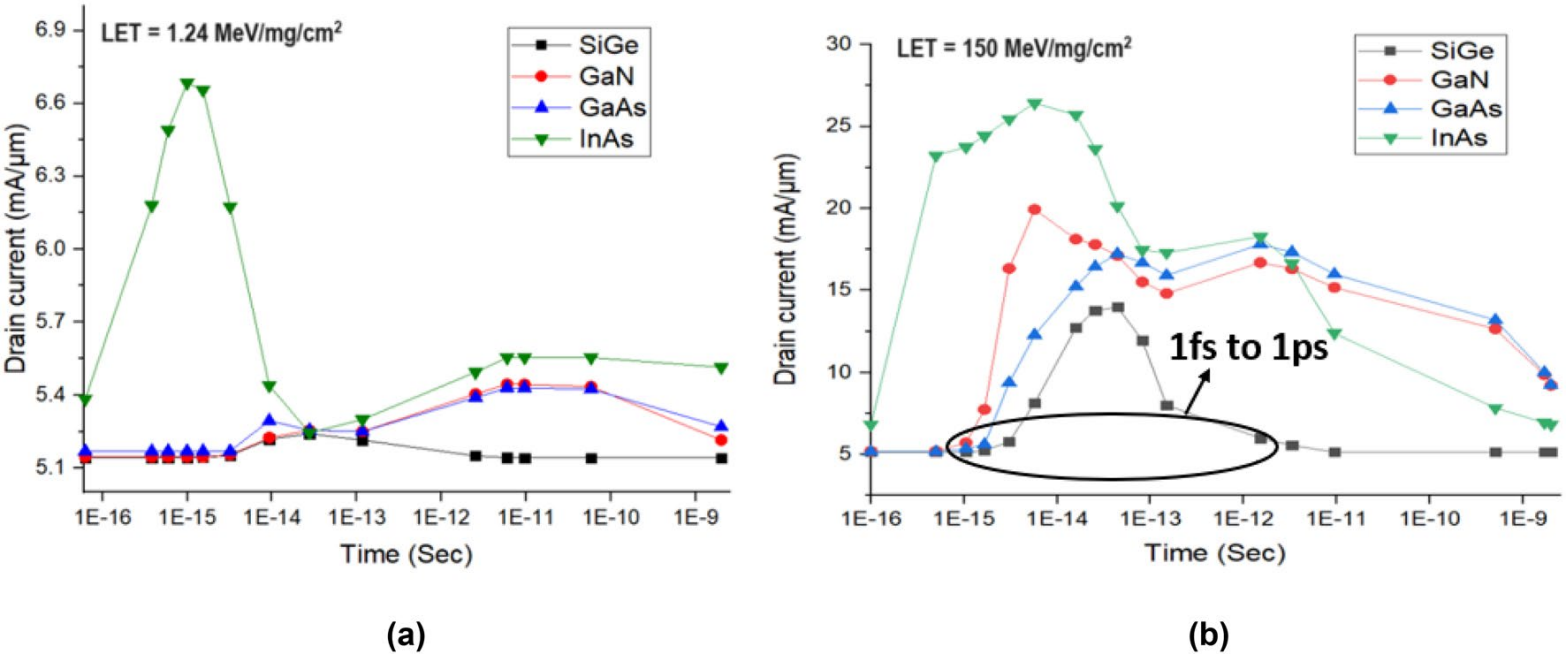
Drain current due to heavy ion irradiation for (**a**) lower dose and (**b**) higher dose of LET.

The change in drain current for different time instants and the change corresponding to a lower and higher dose of LET is plotted in Fig. 10. It can be seen from Figs. 8, 10 that the variation of drain voltage and current show inverse trend with each other for both lower and higher dose of LET. Figure 10a shows that for a lower dose of radiation, SiGe, GaN and GaAs shows lesser I_peak_ and is found to be approximately equal to I_ON._ From Fig. 10b it can be inferred that for a higher dose of LET all the material shows increased I_peak_ than I_ON_ but SiGe shows lesser I_peak_ than other materials. In SiGe based HJLTFET, the transient peak decreases very fast from 1 fS to 1 pS whereas other material shows a wider transient peak and reaches the initial drain current at 1 nS showing lesser sensitivity towards radiation^21^.

**Figure 11:**
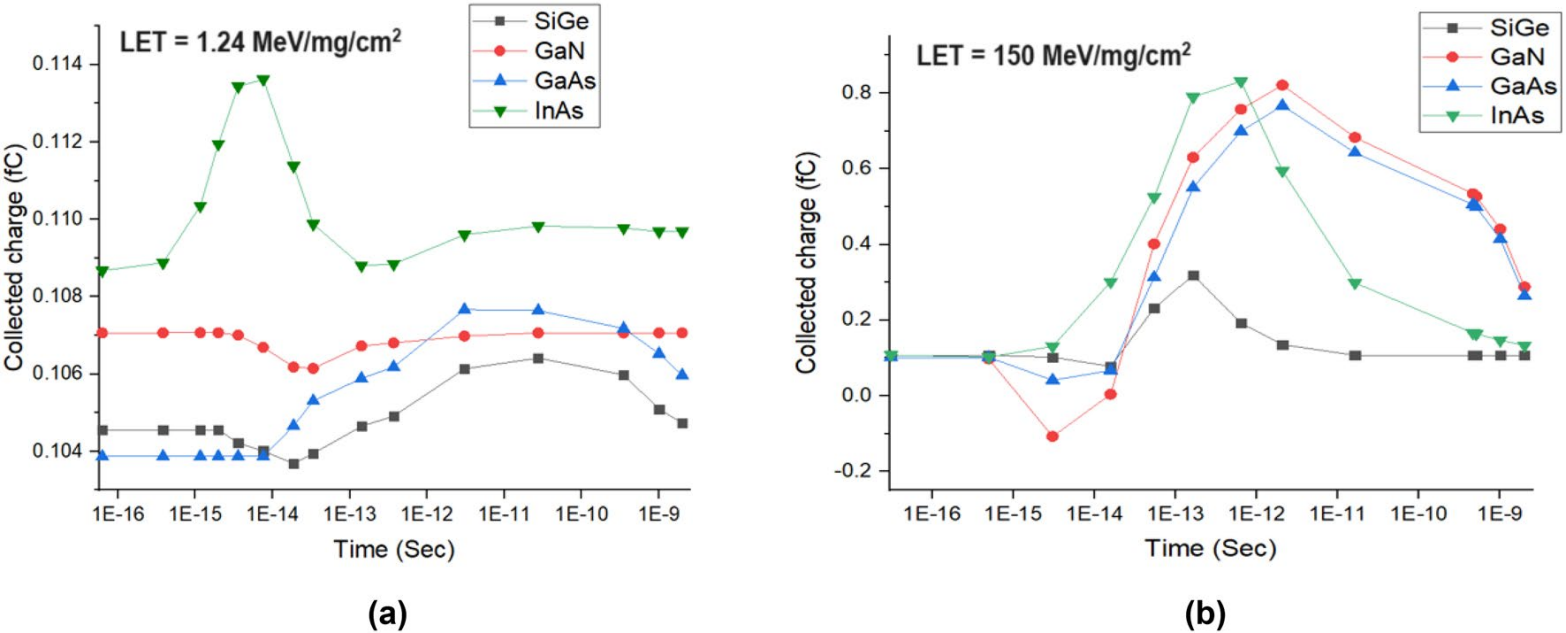
Collected charge due to heavy ion irradiation for (**a**) lower dose and (**b**) higher dose of LET.

The variation of collected charge with respect to different time instants for lower and high dose of LET for different materials is plotted in Fig. 11. It can be seen from Fig. 11a that for a lower dose of LET, Q_C_ of InAs is higher followed by SiGe, GaAs and GaN. It can be observed from Fig. 11b that for higher dose of LET, Q_C_ is found to be higher for InAs and less for SiGe. From Fig. 11a,b it can be inferred that Q_C_ increases with an increase in dose and it can be reasoned out with the drain current changes as shown in Fig. 10^21^.

**Figure 12:**
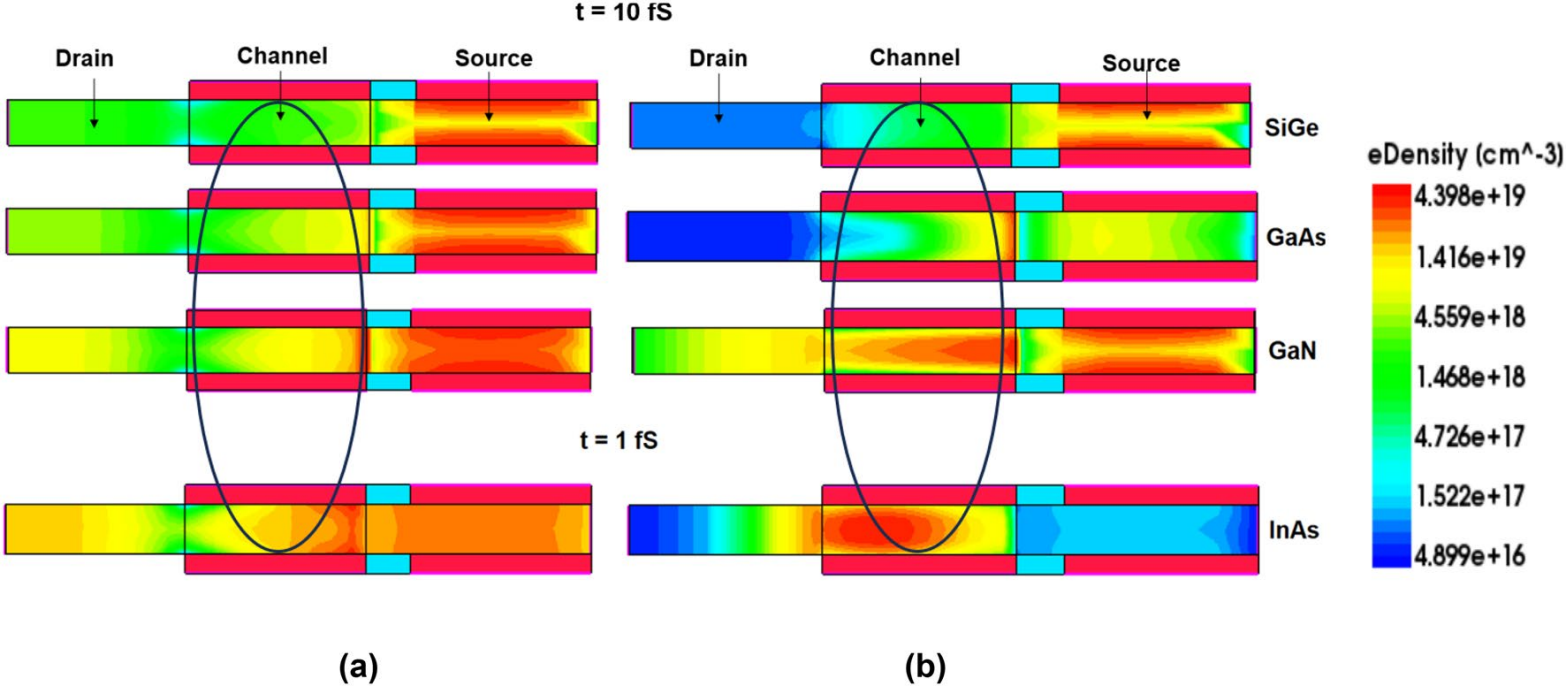
2-D contour profile of electron density for (**a**) low LET value = 1.24 MeV/mg/cm^2^ and (**b**) high value of LET = 150 MeV/mg/cm^2^.

Though the ion strike is made at different locations for different materials, the intensity of electrons is seen clearly only at the centre of the channel. This can be evident by observing the electron density contour plot at the centre of the channel. The 2D contour profile of electron density for lower and higher dose of LET at peak time instant is shown in Fig. 12a,b. The rounded region in the plot gives the maximum intensity of electrons during the ion strike. It can be seen from Fig. 12a that for lower dose of LET values, the density of electrons reaches its peak of ~ 6 × 10^18^ cm^−3^ (yellowish orange) for InAs whereas SiGe shows the least value of ~ 1 × 10^18^ cm^−3^ (green). Similarly, from Fig. 12b, it can be seen that the electron density reaches a peak value of ~ 4.4 × 10^19^ cm^−3^ (red) for InAs whereas SiGe has a lesser electron density of ~ 1 × 10^18^ cm^−3^ (green). In both the cases of LET values, the electron density of GaAs and GaN takes the intermediate values and thus their sensitivity lies in between InAs and SiGe.

**Figure 13:**
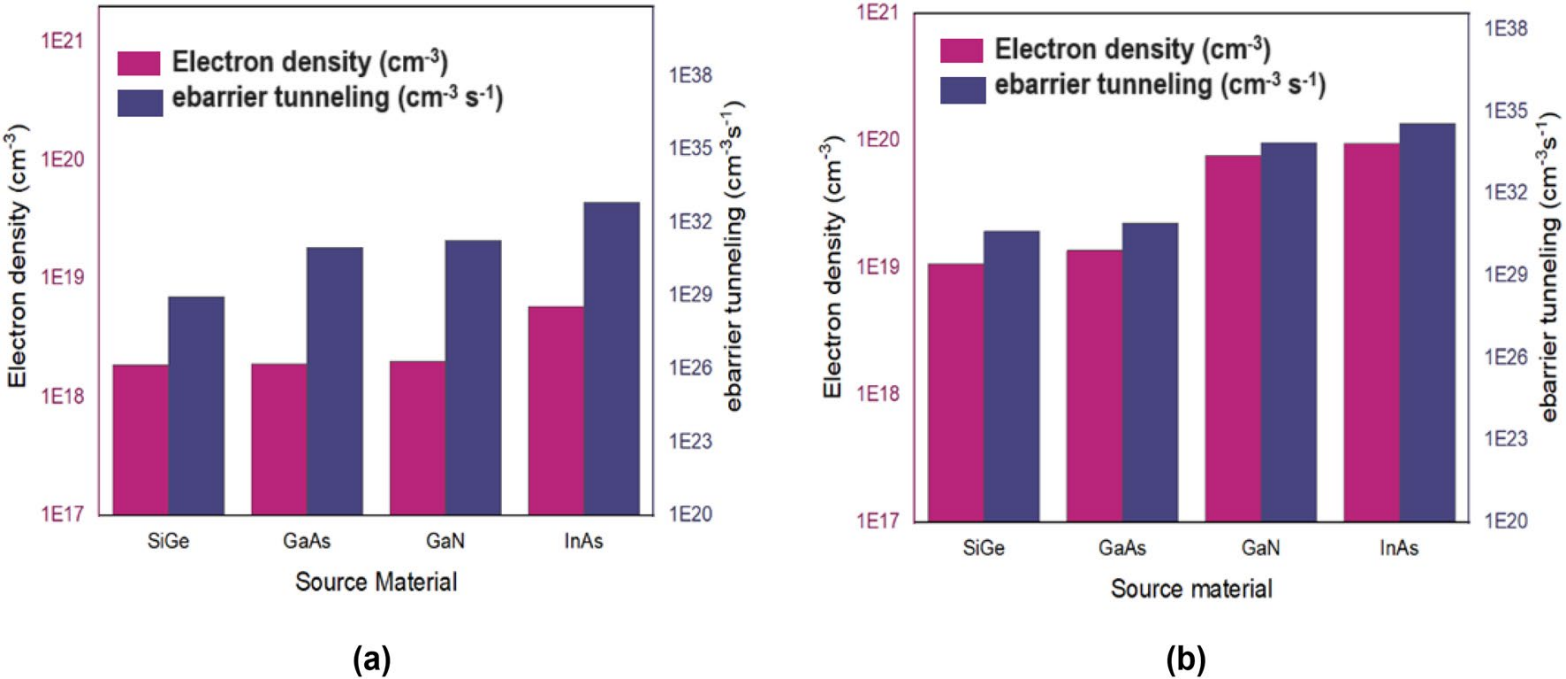
Electron density and ebarrier tunneling for different materials at peak time for (**a**) LET = 1.24 MeV/mg/cm^2^, (**b**) LET = 150 MeV/mg/cm^2^.

The behaviour of the drain current and collected charge for various materials can be better understood by observing the values of electron density and ebarrier tunneling metrics. Figure 13 give the absolute values of electron density and ebarrier tunneling for both lower and higher dose of LET values at the peak time where the drain current reaches its peak value. It can be seen from Fig. 13a that for lower LET dose, electron density remains lower for SiGe, GaN and GaAs due to lesser tunneling occurring at the S/C interface. For InAs, the electron density is higher because of increased tunneling at the D/C interface^54^. Figure 13b shows that for higher dose of LET, electron density and tunneling are higher for InAs and found to be lesser for SiGe.

From all of the above results, it can be observed that for all doses of LET, I_peak_ of InAs is found to be higher than I_ON_ as electron density and ebarrier tunneling are higher. So, it can be inferred that heavy ion sensitivity for InAs is more comparatively than any other materials. On the other hand, it can be observed that SiGe is found to be less sensitive than other materials due to reduced ebarrier tunneling and electron density at all values of LET. Materials with higher electron affinity have higher drain current and this occurrence is observed in our study also^55,56^. Hence the sensitivity of the devices would be higher due to the higher electron affinity of materials whereas materials with lesser electron affinity would possess the least sensitivity towards heavy ion strike.

**Figure 14:**
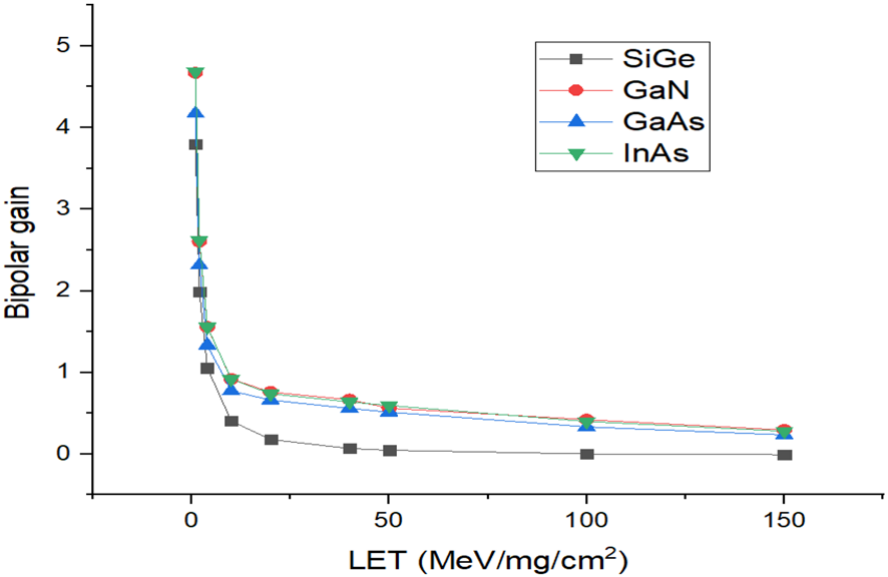
Bipolar gain of HJLTFET for different values of LET.

Another important metric, bipolar gain is calculated for varying LET values as shown in Fig. 14. It can be seen that when LET increases, β reduces because of the higher injection regime of the bipolar transistor^23^. It can be found from Fig. 14 that bipolar gain for SiGe is smaller in comparison with GaN, GaAs and InAs due to less Q_c_ of SiGe compared to other materials^24^. Table 2 presents the performance comparison of HJLTFET with the various source materials. It can be seen from Table 2 that I_peak_ and ΔV_th_ of SiGe is lower than other source materials. Table 3 provides an insight into the performance achieved by the proposed Si/SiGe based HJLTFET against the previous state-of-art devices. It could be observed that for a very less leakage current, HJLTFET achieves high peak current which improves collected charge and ultimately higher bipolar gain.

**Table 2:** Performance comparison of HJLTFET with the various source material combination.

Source material	I_peak_ (mA/µm)		ΔV_th_ (V)	
	Low dose	High dose	Low dose	High dose
SiGe	5.265	16.95	0.004	0.18
GaAs	5.43	17.8	0.0056	0.25
GaN	5.445	19.9	0.0058	0.29
InAs	6.69	26.45	0.0307	0.42

**Table 3:** Performance comparison of HJLTFET with the various state-of-art devices.

Device	I_ON_ (A)	I_OFF_ (A)	SS (mv/dec)	I_peak_ (A)	Q_C_ (fC)	β
Wang et al.^19^	10 × 10^−6^	~ 10^−10^	–	11 × 10^−6^	0.1	2
Munteanu et al.^21^	0.1 × 10^−6^	~ 10^−10^	71	0.75 × 10^−6^	0.5	8
Wang et al.^23^	10^−6^–10^−5^	~ 10^−17^	60	7.63 × 10^−5^	0.4	–
Dubey et al.^24^	~ 10^−4^	~ 10^−11^	–	3.8 × 10^−6^	0.01	0.001
HJLTFET (Si/SiGe) (our proposed work)	10.29 × 10^−3^	2.86 × 10^−18^	60.1	10.53 × 10^−3^	0.103	3.6

## Conclusion

In this study, HJLTFET is designed with silicon in drain and channel region. In contrast, different materials SiGe, GaAs, GaN and InAs are used in source region. HJLTFET is studied for its radiation tolerance with matched I_ON_ for all materials. To find the most sensitive region of the device, a heavy ion strike is performed at lower and higher dose of LET values on all regions. It is found that the D/C interface is sensitive to InAs and S/C interface is sensitive to SiGe, GaAs and GaN. It is noted that Q_C_ of SiGe is 72.7% lesser than InAs. It is observed that for SiGe, the parameters, I_peak_ and Q_C_ show little sensitivity due to lesser ΔV_th_, reduced electron density and tunneling whereas InAs show high sensitivity for all doses. It could also be observed that bipolar gain is lesser for SiGe when compared with GaN, GaAs and InAs. Thus, it can be concluded that Si/SiGe based HJLTFET could be the most promising device suitable for radiation hardening applications in near future.

## Data Availability

All data generated or analyzed during this study are included in this published article.
